# Affective Touch Enhances Self-Face Recognition During Multisensory Integration

**DOI:** 10.1038/s41598-017-13345-9

**Published:** 2017-10-10

**Authors:** Elena Panagiotopoulou, Maria Laura Filippetti, Manos Tsakiris, Aikaterini Fotopoulou

**Affiliations:** 1 University College London, Research Department of Clinical, Educational & Health Psychology, London, UK; 2 Royal Holloway, University of London, Department of Psychology, Egham, Surrey, UK

## Abstract

Multisensory integration is a powerful mechanism for constructing body awareness and key for the sense of selfhood. Recent evidence has shown that the specialised C tactile modality that gives rise to feelings of pleasant, affective touch, can enhance the experience of body ownership during multisensory integration. Nevertheless, no study has examined whether affective touch can also modulate psychological identification with our face, the hallmark of our identity. The current study used the enfacement illusion paradigm to investigate the role of affective touch in the modulation of self-face recognition during multisensory integration. In the first experiment (N = 30), healthy participants were stroked on the cheek while they were looking at another face being stroked on the cheek in synchrony or asynchrony with affective (slow; CT-optimal) vs. neutral (fast; CT-suboptimal) touch. In the second experiment (N = 38) spatial incongruence of touch (cheek vs. forehead) was used as a control condition instead of temporal asynchrony. Overall, our data suggest that CT-optimal, affective touch enhances subjective (but not behavioural) self-face recognition during synchronous and spatially congruent integration of different sensations and possibly reduces deafference during asynchronous multisensory integration. We discuss the role of affective touch in shaping the more social aspects of our self.

## Introduction

While classic views on interoception define it as the “perception of the body from within”, according to a more recent notion of interoception the perception of the physiological state of the body involves both sensations from within the body (e.g. cardiac and respiratory functions, thirst, digestion), as well as sensations whose stimuli are typically located outside the body (e.g. taste, pain, affective touch)^1^, see^2^ for discussion of the definition. Sensory pleasure on the skin, i.e. affective touch, is thought to be coded by specialised, slow-conducting, unmyelinated peripheral nerve fibers, known as C tactile afferents^3,4^, which are found in hairy skin^5,6^. The optimal stroking velocities at which they fire vigorously are 1–10 cm/sec^7^ and the activation of CT-afferent fibers correlates with subjective ratings of pleasantness, indicating that CT-afferents may constitute a peripheral ascending pathway for pleasant tactile stimulation^7^. Research has shown that CT-afferents may project via thalamic pathways to the posterior insular cortex^3,8^ (but see^9^), conferring thus the affective value of this type of touch.

Recent evidence suggests that affective touch may be involved in the construction and maintenance of body ownership^10–13^ which is defined as the psychological sense that the physical body “belongs to me”^14^. Over the last 20 years, psychophysical and computational research has shown that the sense of body ownership relies on the integration of information from different sensory modalities, the so-called, multisensory integration^15^. Well-known paradigms such as the “rubber hand illusion” (RHI^15,16^;) and the “full body illusion”^17,18^ have demonstrated that temporal and spatial congruence between felt and seen sensory events gives rise to feelings of body ownership, even for plastic or virtual body parts. Importantly, other aspects of the bodily self, such as the ability to recognise our own face, appear susceptible to change through synchronous, multisensory stimulation^19–21^. Self-face recognition is considered as an index for self-awareness^22–24^, thought to be supported by both off-line stored information on one’s face appearance^25,26^ and on on-line multisensory integration mechanisms^26,27^. It was only recently that the role of multisensory integration in self-face recognition was tested for the first time^19^. Participants were stroked on the face while they were watching a video with another face being stroked in synchrony or asynchrony (1s delay) and they performed a self-recognition task before and after this interpersonal visuo-tactile stimulation. The results showed that after synchronous, but not asynchronous stimulation, participants accepted as self-stimuli faces that were significantly more morphed towards the other face. Similar effects were reported in the description of this “enfacement illusion”^20^, while there is also evidence that this blurring of self-other boundaries extends beyond body perception to a more conceptual merging between self and other, by affecting social cognition processes^21^. Moreover, research has shown that synchronous interpersonal multisensory stimulation influences affective judgements of the other face, including ratings of greater attractiveness and trustworthiness^28^.

However, to the best of our knowledge, the opposite relation, namely how affective experiences may influence self-face recognition has not been investigated. Even in the case of body ownership, the majority of studies to date have focused on how exteroceptive signals such as vision and discriminatory touch are integrated, while the role of interoceptive signals in body ownership has been investigated only recently. For instance, in two virtual reality studies^29,30^, visual feedback of participants’ own heartbeat was provided ‘on-line’ (i.e. during the virtual reality tasks) by means of a flashing virtual body or hand in synchrony or out-of-synchrony with the participants’ own heartbeats, with the synchronous condition increasing self-identification with the virtual body^29^ and embodiment of the rubber hand^30^, respectively. Thus, when interoceptive signals are artificially provided also in the visual domain, vision seems capable of ‘capturing’ interoception, leading to enhanced down regulation of proprioception as in the classic RHI paradigm. Most importantly for the present study, affective (CT-optimal) touch has been found to enhance the experience of owning a rubber hand more than neutral (CT-suboptimal) touch, in both subjective report^10,12,13^ and in implicit, behavioural measures^11^ (but see^31^ for mixed results). Such findings suggest a strong link between affective touch and body awareness^32–34^. More generally, it has been suggested that the core affective aspects of selfhood are shaped by embodied interactions with other people throughout the lifespan and starting with affective touch experiences in early infancy^34^. Nevertheless, the potential (developmental) role of affective touch in self-formation and self-other boundaries remains an understudied scientific question. While there is the above evidence that affective touch modulates the experience of body ownership, no study has tested whether increasing affectivity during the process of multisensory integration would enhance effects of recognition of our own face, an important aspect of personal and social identity.

To this end, the present study used the enfacement illusion paradigm to investigate for the first time the role of affective touch in the multisensory modulation of self-face recognition in two experiments. In the first experiment, participants were stroked on the cheek while they were looking at another unfamiliar face being stroked on the cheek in synchrony or asynchrony with affective (slow; CT-optimal) vs. neutral (fast; CT-suboptimal) touch. The second experiment was identical to the first but instead of using asynchrony, spatial incongruence of touch (cheek vs. forehead) was introduced as an alternative control condition. In both experiments, participants were asked to perform a self-recognition task before and after the interpersonal visuo-tactile stimulation in which they were presented with a movie showing their face morphing in 1% incremental steps into the other face, or vice versa, and they were instructed to stop the movie at the point where they felt that the face began to look more like the face it was morphing into. They also completed a questionnaire (see details in Methods) capturing their subjective experience. In line with previous evidence, we predicted higher levels of enfacement for affective (slow) vs. neutral (fast) touch when the stroking was synchronous (Experiment 1) and spatially congruent (Experiment 2).

## Experiment 1

### Results

#### Subjective Enfacement

Overall Enfacement: *Composite score of Self-identification, Similarity and Affect*.

**Figure 1:**
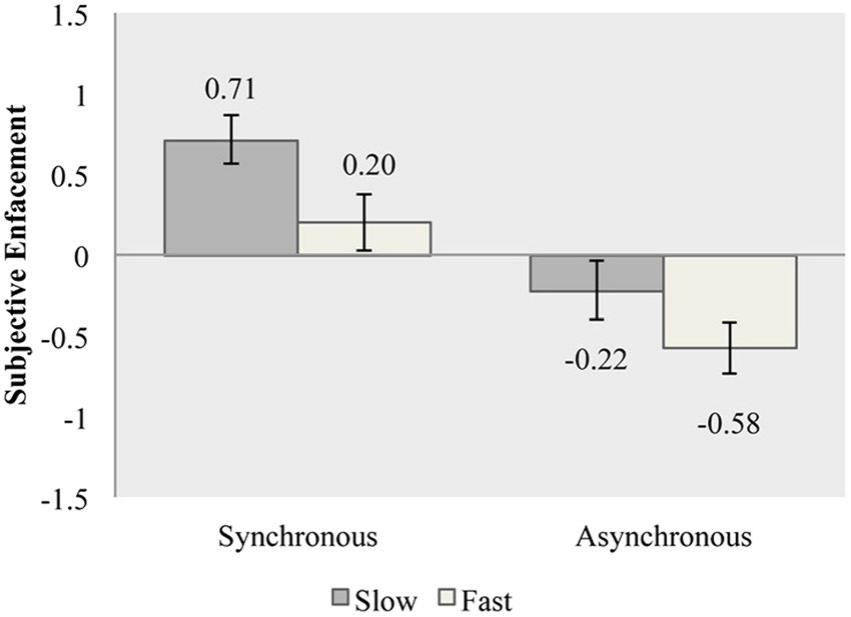
Means for overall subjective enfacement in Exp. 1. Higher scores indicate greater enfacement. Error bars denote standard errors.

A 2 × 2 ANOVA revealed a significant main effect of “synchrony” (F (1,29) = 40.53, *p* < 0.001, η^2^ = 0.583) with synchronous stroking (mean = 0.456, SE = 0.125) producing higher levels of subjective enfacement as compared to asynchronous stroking (mean = −0.400, SE = 0.142), which confirmed the presence of the illusion. A significant main effect was also found for “stroking velocity” (F (1,29) = 5.87, *p* = 0.022, η^2^ = 0.168) with slow stroking (mean = 0.247, SE = 0.146) producing higher levels of subjective enfacement as compared to fast stroking (mean = −0.192, SE = 0.148). The interaction between “synchrony” and “stroking velocity” was not significant (F (1,29) = 0.811, *p* = 0.375, η^2^ = 0.027), (Fig. 1).

Sub-component analysis: The same pattern of results was found for the individual subcomponents of the questionnaire, with synchronous stroking, as compared to asynchronous, producing higher levels of both self-identification [F (1,29) = 36.03, *p* < 0.001, η^2^ = 0.554] and similarity [F (1,29) = 24.07, *p* < 0.001, η^2^ = 0.454] and slow stroking, as compared to fast, also producing higher levels of self-identification [F (1,29) = 4.86, *p* = 0.036, η^2^ = 0.144] and similarity [F (1,29) = 6.98, *p* = 0.013, η^2^ = 0.194]. With regards to the affect component, synchronous stroking led to higher levels of affect towards the other face [F (1,29) = 6.32, *p* = 0.018, η^2^ = 0.179]. The effect of stroking velocity was not significant but we note a trend of more affect following slow stroking as compared to fast stroking [F (1,29) = 3.42, *p* = 0.075, η^2^ = 0.106]. All interactions between synchrony and stroking velocity were non-significant.

#### Self-recognition task

**Figure 2:**
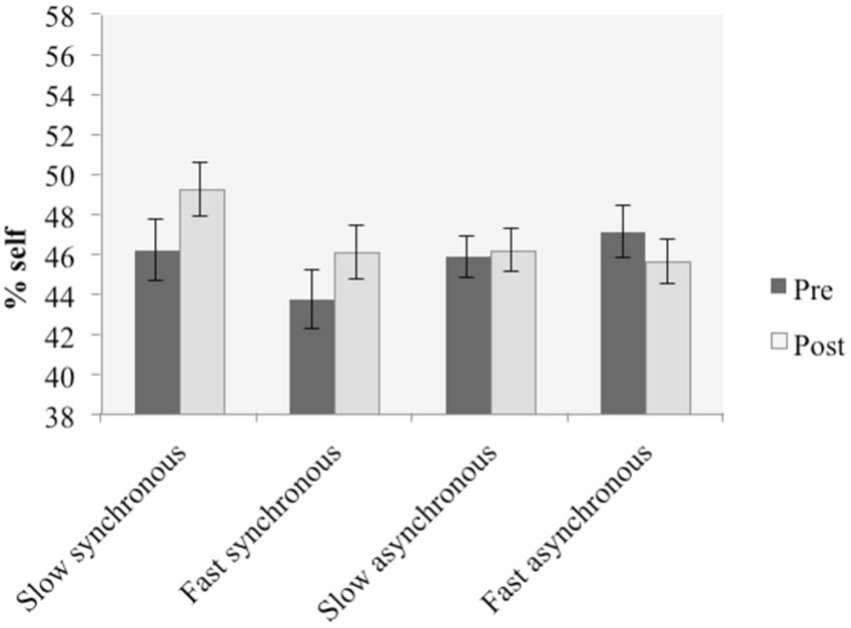
Means for percentage of frames containing ‘self’ in the different conditions. Error bars denote standard errors.

The analysis revealed a significant main effect of “judgment” (F(1,29) = 4.81, *p* = 0.036, η^2^ = 0.142). The two-way interaction between “synchrony” and “judgment” was significant (F(1,29) = 4.41, *p* = 0.045, η^2^ = 0.132), as well as the two-way interaction between “synchrony” and “stroking velocity” (F(1,29) = 7.52, *p* = 0.010, η^2^ = 0.206). Bonferroni-corrected post hoc tests revealed that the percentage of “self” in the average frame significantly increased in post-tests when the stimulation was synchronous (t(20) = −2.72, *p* = 0.011, *d* = 0.41) but not asynchronous (t(29) = 0.719, *p* = 0.478, *d* = 0.11). Moreover, slow stimulation led to a significantly higher percentage of “self” in the average frame as compared to fast stimulation, only when the touch was synchronous (t(29) = 2.59, *p* = 0.015, *d* = 0.41) but not asynchronous (t(29) = −0.349, *p* = 0.729, *d* = 0.06) (Fig. 2).

#### Pleasantness Ratings

To establish whether slow touch was perceived as significantly more pleasant that fast touch (a manipulation check), we examined the main effect of “stroking velocity” on pleasantness ratings. A Wilcoxon signed ranked test confirmed that participants perceived slow stroking (median = 1.25, IQR = 1.63) as significantly more pleasant than fast stroking (median = −0.25, IQR = 2.00, Z = −4.45, *p* < 0.001, r = −0.812).

## Discussion

The first experiment set out to investigate the modulation of self-face recognition by affective touch using asynchrony as a control condition. Participants were stroked on their cheek while they were watching an unfamiliar face being stroked on the cheek in synchrony or asynchrony with slow (3 cm/s) vs. fast (18 cm/s) velocity. We predicted higher levels of enfacement for slow vs. fast velocity when the stimulation was synchronous.

As expected, we found that slow touch (3 cm/s) was perceived as more pleasant than fast touch (18 cm/s). This suggests that our touch manipulation was successful. With regards to subjective enfacement (as captured by the enfacement questionnaire), we found that synchronous stimulation led to higher levels of enfacement as compared to asynchronous stimulation, confirming the important role of multisensory integration in self-face recognition^19–21,28^. Yet, we also provide the first direct evidence that the velocity of touch is crucial, given that affective (slow) touch led to significantly higher levels of overall subjective enfacement as compared to neutral (fast) touch. Interestingly, and contrary to our predictions, no interaction between synchrony and stroking velocity were found. Thus, subjective enfacement was present when the stimulation was synchronous, i.e. positive ratings, and these appeared to increase further when slow, affective touch was applied. By contrast, asynchronous stimulation led to negative ratings, yet these were less negative in slow than in fast touch. Thus, affective touch increased feelings of enfacement and decreased feelings of non-enfacement. We had predicted the former effect of affective touch on synchronous stimulation but we had not predicted the effect of affective touch on asynchronous stimulation. Taken together, our results suggest that synchrony and affective touch may have orthogonal effects on self-face recognition during the enfacement illusion (see below for potential mechanisms behind these effects and the rationale behind our second experiment).

The same pattern of results described for the overall subjective enfacement was also seen for the individual sub-components, self-identification and similarity. While previous research has shown that even when we feel dissimilar to others, shared multisensory experiences can change the self-other boundaries leading to increased similarity^19,21^, in this study we demonstrated that when those synchronised sensory experiences are affective in nature, perceived similarity and identification with others increased even more when the stimulation was synchronous and decreased less when the stimulation was asynchronous. It therefore appears that affective touch in the context of multisensory integration can alter aspects of the selfhood, i.e. self-other boundaries^34^, giving rise to a “like-me” nature of others. According to developmental models, this “like-me” nature is the starting point for social cognition and intersubjectivity^35,36^.

With regards to the self-recognition task, we confirmed the presence of the illusion, by finding a two-way interaction between synchrony and judgment. That is, synchronous stimulation, but not asynchronous, led to a significant change in self-recognition. We also found a significant two-way interaction between synchrony and velocity, but this was not significant when baseline scores were taken into account in the three-way interaction. Therefore, the results show that affective touch had no significant effect on behavioural self-face recognition.

More generally, recent studies have shown that both synchrony and asynchrony can have effects on multisensory integration and on body ownership and these may be caused by partly independent mechanisms^37,38^. Specifically, while synchrony may be a necessary condition for multisensory integration, asynchrony may cause more than just ‘non-integration’ of sensory modalities^37^. Indeed, the intersensory conflict arising from asynchronous stimulation has been found to underlie unpleasant feelings of “deaffererence”, a sensation that one’s own limb is felt as numb and less vivid^39^. Thus, asynchrony, the most commonly used control condition in multisensory integration paradigms, may not actually be perceived as a neutral, baseline condition.

In this experiment, we demonstrated that affective touch, as a specialised interoceptive modality with positive valence, leads to increased subjective enfacement (as compared to neural touch) after synchronous tactile stimulations, in line with previous research^10,13^, which has shown that the experience of owning a rubber hand is enhanced when the touch is slow and synchronous. Yet, what we also demonstrated for the first time is that slow, affective touch also leads to less subjective “resistance” to the illusion in asynchronous conditions. We suggest that this reduction may be explained as a reduction of subjective “deafference” by affective touch during asynchronous stroking.

To validate our conclusions regarding our main aim, namely the role of affective touch in self-face recognition, we conducted a second experiment using spatial incongruence as a control condition in order to understand whether our results were due to general affectivity or potentially orthogonal mechanisms of affective touch and temporal synchrony effects on self-face recognition. We chose spatial incongruence as a control condition given that there is no evidence that it causes the same degree of “deafference” as asynchrony, and also because it controls for the general effect of increased attention during synchronous interpersonal visuo-tactile stimulation^40^. In other words, synchronous stimulation ensured comparable levels of attention, whereas the congruence of the location was used to selectively induce the illusion or not. In the second experiment, participants were touched on a congruent or incongruent location (cheek vs. forehead) with affective (slow; CT-optimal) vs. neutral (fast; CT-suboptimal) touch. We expected slow, affective touch to enhance enfacement as compared to fast, neutral touch for spatially congruent stimulation, but not to reduce “resistance” to the enfacement for spatially incongruent stimulation.

## Experiment 2

### Results

#### Subjective Enfacement

Overall Enfacement: *Composite score of Self-identification, Similarity and Affect*.

**Figure 3:**
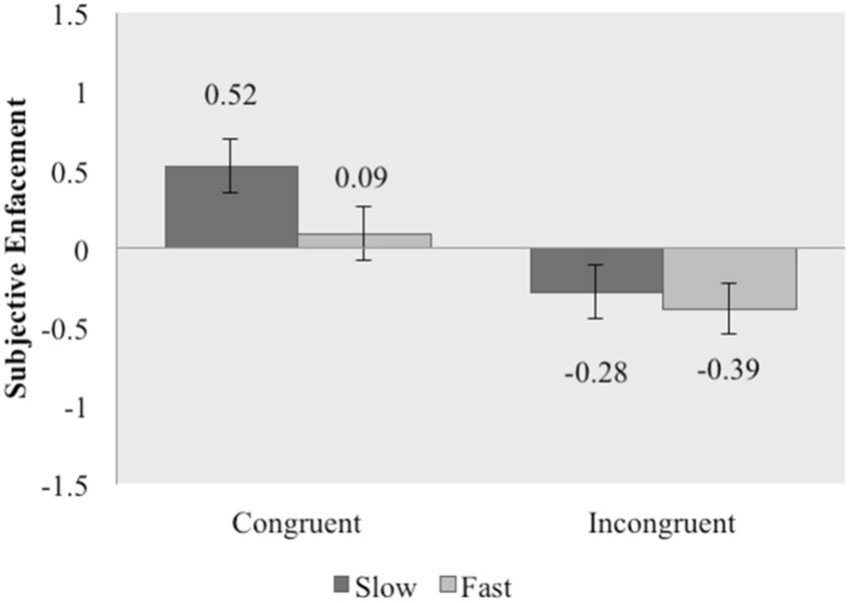
Means for overall subjective enfacement in Exp. 2. Higher scores indicate greater enfacement. Error bars denote standard errors.

A 2 × 2 repeated ANOVA revealed a significant main effect of “spatial congruence” (F (1,37) = 31.24, p < 0.001, η^2^ = 0.458) with stroking on a congruent location (mean = 0.304, SE = 0.155) producing higher levels of subjective enfacement as compared to stroking on an incongruent location (mean = −0.336, SE = 0.146), confirming the presence of the illusion. A significant main effect of “stroking velocity” was also found (F (1,37) = 4.83, p = 0.034, η^2^ = 0.116), with slow stroking (mean = 0.120, SE = 0.159) producing higher levels of enfacement as compared to fast stroking (mean = −0.151, SE = 0.146). Furthermore, we found a significant interaction between “spatial congruence” and “stroking velocity” (F (1,37) = 4.21, p = 0.047, η^2^ = 0.102). Bonferroni-corrected post hoc analyses revealed that slow congruent stroking led to significantly higher levels of enfacement as compared to fast congruent stroking (t(37) = 3.12, p =0.004, *d* = 0.51), while the difference between slow incongruent and fast incongruent stroking was not significant (t(37) = 0.780, p = 0.441) (Fig. 3).

Sub-component analysis: For the individual sub-components, we found that self-identification was higher for spatial congruence (F (1,37) = 29.50, p < 0.001, η^2^ = 0.444) and we also noted a trend for slow velocity (F(1,37) = 3.02, p = 0.090, η^2^ = 0.076). The interaction between spatial congruence and velocity was significant (F(1,37) = 4.25, p = 0.46, η^2^ = 0.103). Bonferroni-corrected post hoc analyses revealed that slow congruent stroking led to significantly higher levels of self-identification as compared to fast congruent stroking (t(37) = 2.70, p = 0.010, *d* = 0.44), while the difference between slow incongruent and fast incongruent stroking was not significant (t(37) = 0.069, p = 0.945, *d* = 0.11). With regard to similarity, spatial congruence produced higher levels of similarity as compared to spatial incongruence (F (1,37) = 29.34, p < 0.001, η^2^ = 0.442) and we also noted a trend for slow velocity (F (1,37) = 3.51, p = 0.069, η^2^ = 0.087). The interaction was non-significant. For the affect component we found a trend for spatial congruence (F (1,37) = 3.12, p = 0.086, η^2^ = 0.078) and slow velocity (F (1,37) = 3.48, p = 0.070, η^2^ = 0.086) both leading to higher levels of affect as compared to spatial incongruence and fast velocity respectively. The interaction was non-significant.

#### Self-recognition task

The analysis revealed a significant main effect of “judgment” (F = (1,37) = 11.80, p < 0.001, η^2^ = 0.242), suggesting that, regardless of stroking velocity or spatial congruence, after visuo-tactile stimulation self-other blurring increased. However, the other main effects or interactions were not significant.

#### Pleasantness Ratings

To establish whether slow touch was perceived as significantly more pleasant than fast touch, we examined the main effect of “stroking velocity” on pleasantness ratings (manipulation check). A Wilcoxon signed ranked test confirmed that participants perceived slow stroking (median = 2.00, IQR = 1.63) as significantly more pleasant than fast stroking (median = 1.50, IQR = 1.50, Z = −3.49, *p* < 0.001, r = −0.567).

## General Discussion

Over two experiments, we used the enfacement illusion paradigm to investigate for the first time the role of affective touch in the modulation of self-face recognition. We demonstrate that affective touch, delivered interpersonally according to the properties of the specialised C tactile (CT) afferents and giving rise to subjective feelings of sensory pleasure, played a crucial role in the modulation of self-face recognition during multisensory integration. Specifically, in line with previous studies on CT-optimal touch, our findings confirmed that affective (CT-optimal, slow) touch on the face was perceived as more pleasant than neutral (CT-non optimal, fast) touch^7^. Importantly, in both experiments, when the multisensory stimulation between the two faces was synchronous and spatially congruent, affective touch appeared to lead to higher levels of subjective enfacement of the “other” face as compared to emotionally neutral touch. In Experiment 1, this difference between affective vs. neutral touch was also observed when the stimulation was asynchronous, with affective touch leading to less resistance to subjective enfacement as compared to neutral touch, i.e. less disagreement with the statements of the enfacement questionnaire. We speculate that the positive valence of affective touch has the potential to reduce the “deafference” (e.g. unpleasant, numb feelings about the body^39^) caused by the temporal mismatch between felt and seen tactile stimulation. Of course, in order to test this hypothesis regarding the relation between CT optimal touch and deafference, further dedicated study would be needed, for example an experiment in which both factors will be manipulated parametrically and their relation would be thus explored in greater specificity.

As regards the hypothesis and aims of the present study however, in the second experiment, in which participants were only touched synchronously either on congruent or incongruent locations on the face (cheek vs. forehead), we found that affective touch enhanced self-face recognition only in the congruent and not in the incongruent condition, confirming the unique role of affective touch in the processes of multisensory integration that underlie feelings of self-identification. Indeed, affective touch had a different effect on subjective self-face recognition under temporal mismatch (orthogonal effects of tactile affectivity and synchrony on multisensory integration) as compared to spatial mismatch (effect of affectivity was dependent on spatial congruence). As mentioned above, it has been shown that synchrony and spatial congruence during multisensory stimulation lead to a perceptual binding between seen and felt events, while the effects of multimodal asynchronous stimulation may be subject to several other mechanisms^37,38^, including attentional confounds^40^ and “deafference”^39^. Such factors do not seem to apply during multimodal experiences of spatial incongruence, presumably because it is less common (and thus less probable and plausible)^41^ for an individual to be touched in synchrony with another’s body part in a proximal and congruent position, than it is to be touched in synchrony with another’s body part in an incongruent position. This interpretation fits with recent Bayesian predictive coding accounts of self-identification feelings during multisensory integration;^41^ in relation to interoception see^13^. Therefore, the second experiment, which used a control condition without some of the known complexities of “asynchrony”, confirmed that affective touch has a unique effect on self-face recognition during multisensory integration, over and above any general effects of pleasantness or social desirability on subjective judgements.

Yet, our study showed a dissociation between self-report measures (enfacement questionnaire) and behavioural measures (self-face recognition task), given that there was no effect of affective touch on behavioural self-face recognition. This result is in line with recent data from the rubber hand illusion and affective touch, where similar dissociations in the effects of affective touch on the illusion were observed^10–12^. The presence of contradictory findings suggests that future studies should specifically investigate this interesting modulation further.

One limitation of this study is that we did not compare the effects of temporal synchrony and spatial incongruence on the illusion directly and, hence, any comparison is purely qualitative. Moreover, another question raised by this study is whether the modulatory effects of affective touch on enfacement are due to bottom-up CT-afferent signalling or top-down learned expectations conveyed by the “seen” slow touch on the other face^8,42^. Knowing that the CT-afferents are found in hairy skin only (i.e. cheek, forehead) and not on the glabrous skin^5,6^, it would be interesting to compare the effects of slow vs. fast tactile stimulation on both hairy (e.g. cheek) and glabrous sites (e.g. lips) in order to gain insight into the separate involvement of bottom-up mechanisms and top-down expectations of sensory pleasure.

To conclude, this study provides the first direct evidence that embodied affective interactions and particularly affective touch during multisensory integration enhances subjective self-face recognition. These effects were found to be selective to other conditions of multisensory integration and particularly spatial congruence. Thus, the effects of affective touch on self-face recognition do not seem to be explained by general mood or pleasantness effects. Overall, research has shown that affective touch may have a great impact on our physical and emotional well-being. Social, affective touch may serve as a homeostatic regulator^34^, a “stress buffer”, regulating the body’s responses to acute stressors^43^, as well as a regulator of fundamental bodily emotions, such as pain^44,45^. The present study provides evidence that CT-optimal affective touch may also affect our bodily self and ultimately determine how one perceives the boundaries of his/her own body^34^. Increasing embodied affectivity appears to have a potent role in the formation of the bodily boundaries of the self, by allowing identification of the psychological self with a physical body and, more specifically, a face, which is considered a hallmark of our identity.

## Methods

### Experiment 1

#### Participants

Thirty right-handed healthy females (age 25.16 ± 3.13SD years) were recruited from UCL Psychology Subject Pool and took part in a single 1-hour session for course credit or £10. Participants had no known physical and mental illness and no skin diseases. Signed informed consent was obtained from all participants prior to their participation. The study was approved by the Ethics Committee of the Research Department of Clinical, Educational and Health Psychology, University College London. The experiment was performed in accordance with relevant guidelines and regulations.

#### Design

The experiment employed a 2 × 2 within-subjects design with two factors: 1) Synchrony (synchronous vs. asynchronous) and 2) Stroking Velocity (slow, affective vs. fast, neutral). The dependent measures were: a) a self-recognition task as a behavioural measure of the illusion and b) an enfacement questionnaire capturing the subjective experience of the illusion (see Materials section for details). As a manipulation check, we asked participants to rate how pleasant the touch was.

#### Materials

Enfacement movies: For the induction of the illusion, enfacement movies were created displaying an unfamiliar face (same sex, same age +/−5 years; see^28^) being stroked on the cheek with a cosmetic-like soft brush. Each stroke covered a distance of 6cm on the cheek and there were two stroking velocities; slow (3 cm/s) and fast (18 cm/s). The stimulation time across slow and fast stroking was kept constant^10,13^. A resting period interleaved each stroke. For slow touch, there were 2 sec of stimulation followed by 2 sec of rest, while for fast touch there was 1 sec of stimulation followed by 1 sec of rest. Each movie lasted 120 sec with 60 sec of tactile stimulation and 60 sec of rest. The mismatch between visual and tactile stimulation in the asynchronous conditions was 1 second, hence the amount of stimulation across synchronous and asynchronous was kept constant. Two different unfamiliar faces were used in the experiment, so that half of the participants saw Face 1 for slow and Face 2 for fast touch and vice versa.

Morphing movies: A digital photograph of the participant was taken at the beginning of the experimental session. The participant’s face in the photograph was mirror-transposed, converted to greyscale, and all non-facial attributes were removed (e.g. background, hear, ears) with GNU Image Manipulation Program (GIMP). A computerized morphing procedure implementing a mesh warping algorithm (Abrasoft Fantamorph) was used to merge each participant’s face with the unfamiliar face (Face 1 and 2) in 1% steps resulting in 100 frames with graded blending of the facial features of the two faces. For each participant, four morphing movies were created since there were two unfamiliar faces (Face 1 and 2) and two directions: from 100% self to 0% self (“*self to other”* direction) and from 0% self to 100% self (*“other to self”* direction). Each movie lasted 33 seconds and contained 100 frames.

**Figure 4:**
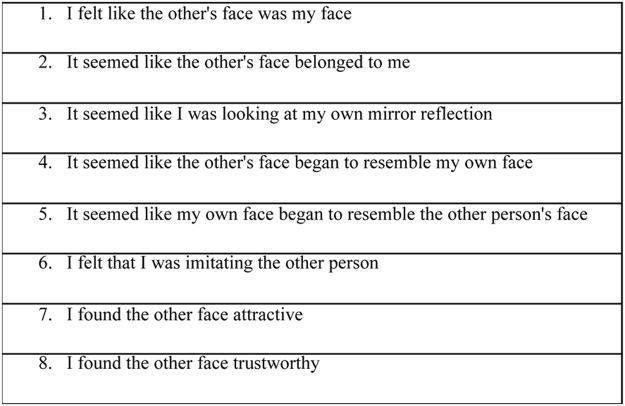
Enfacement Questionnaire.

Enfacement questionnaire: After each stimulation session, participants were also asked to complete an enfacement questionnaire to capture the subjective experience of the illusion. The questionnaire contained 8 items presented in a random order (7-point Likert-type scale; −3, strongly disagree; +3, strongly agree) (Fig. 4). The statements were selected from previous studies of multisensory-induced body illusions (see^28^). Based on a principal component analysis (PCA)^28^, the questionnaire used consisted of three sub-components: *self-identification*, that is the extent to which participants feel that the other’s face is theirs (items 1–3, 6); *similarity*, that is the extent to which participants perceive the other’s face as similar to theirs (items 4,5); and *affect*, that is the extent to which participants judge the other’s face as attractive and trustworthy (items, 7,8). The overall subjective enfacement score was obtained by averaging these three subcomponent scores.

#### Procedure

**Figure 5:**
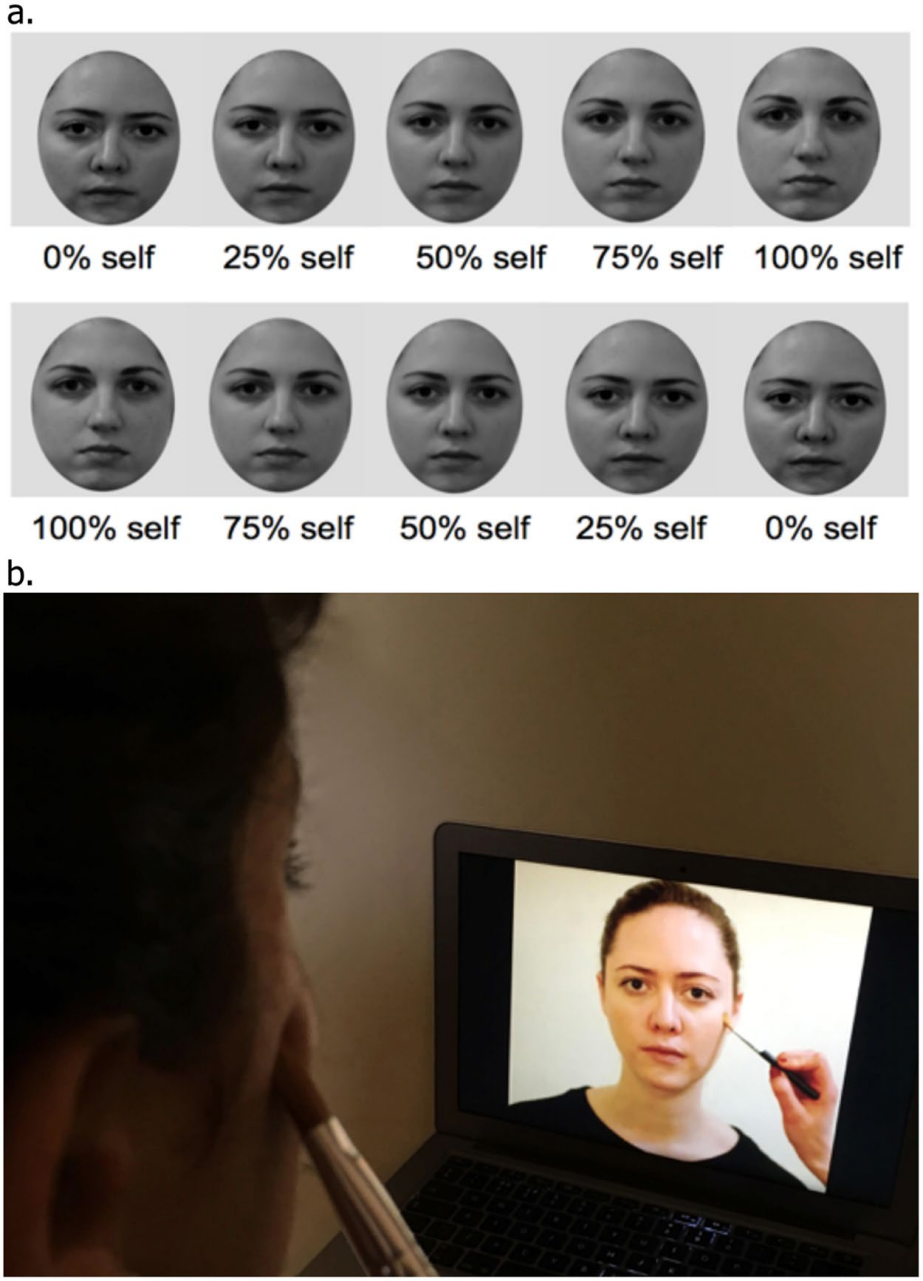
Illustrative example of the morphing procedure with direction of morphing (from “other to self” and from “self to other”) displayed in the two types of movies (Fig. 5a) and experimental set-up during interpersonal visuo-tactile stimulation (Fig. 5b).

**Figure 6:**
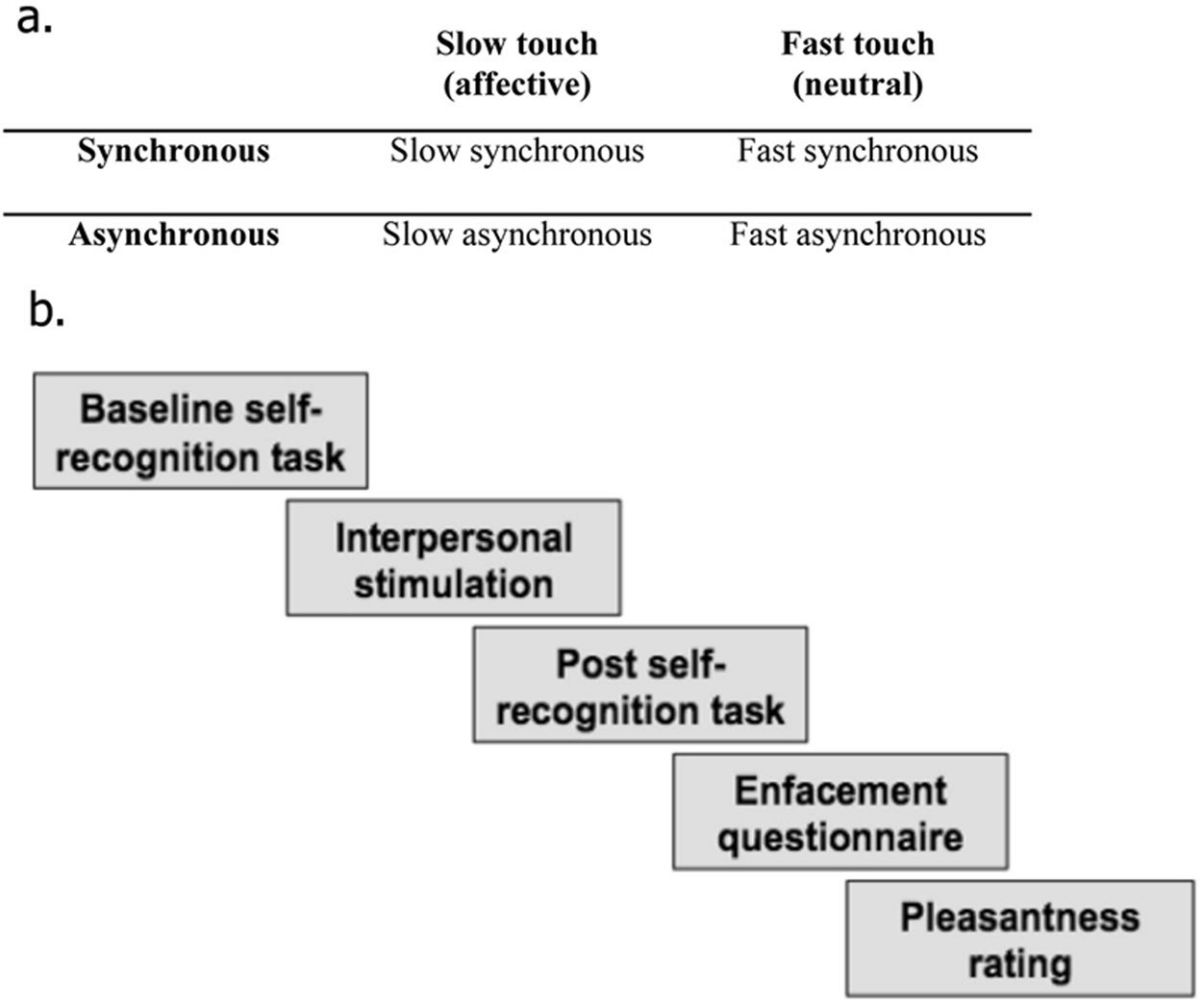
Four different conditions (blocks) (Fig. 6a) and experimental procedure per block (Fig. 6b).

Computer-generated stimulation was controlled by a customized software program (Presentation software, Neurobehavioral Systems Inc.) and presented on the screen, which was placed at a viewing distance of approximately 50 cm. At the beginning of the session, participants were asked to complete the baseline *self-recognition task* watching the morphing movie (Fig. 5a). For the “other to self” direction of morphing, participants were asked to press the space key, with their right index finger, as soon as they perceived the face to look more like “self” than “other”. For the “self to other” direction movies, participants were asked to press the same key when they perceived the face to look more like “other” than “self”. As soon as the participants pressed the key, the movie stopped and the number of seconds at which the movie was stopped was recorded each time. Participants received prior training on this task, by watching movies where Face 1 was morphing to Face 2 or vice versa. Following the baseline *self-recognition task*, participants were instructed to look at the screen placed in front of them, relax and simply observe the projected (enfacement) movie, which lasted 120 sec. The models in the movie looked straight at the camera with a neutral expression. As soon as the movie began, tactile stimulation was delivered on the participant’s right cheek using the same cosmetic-like soft brush. The experimenter, who was previously trained to deliver synchronous and asynchronous stimulation, manually delivered tactile stimulation on the participant’s cheek on a specular congruent location between both faces (Fig. 5b). Therefore, participants were touched on the same location in four different ways: synchronously with slow velocity (*slow synchronous*), synchronously with fast velocity (*fast synchronous*), with 1-sec-delay and slow velocity (*slow asynchronous*) and with 1-sec-delay and fast velocity (*fast asynchronous*) (Fig. 6a). After the tactile stimulation period, participants performed exactly the same *self-recognition task* as in baseline. At the end of the task, participants completed the *enfacement questionnaire*, followed by the *subjective pleasantness rating* for the tactile stimulation (Fig. 6b). In total, each participant performed 4 blocks (one for each condition). The order of blocks was randomised and there was a two-minute break between blocks, during which participants were asked to look at their face in order to break the illusion (Photobooth application for Mac computers).

#### Data analysis

Statistical analyses were performed using the Statistical Package for the Social Sciences (SPSS) version 23 (IBM, Chicago, IL, USA). For our manipulation check, given that pleasantness ratings were not normal, Wilcoxon signed-rank tests were performed. For the overall subjective enfacement (i.e. average score of enfacement questionnaire) we performed repeated-measures analysis of variance (ANOVA) with Synchrony (synchronous vs. asynchronous) and Stroking Velocity (slow vs. fast) as within-subject factors. We did the same analysis for the individual sub-components of the questionnaire: self-identification, similarity and affect. For the analysis of the self-recognition task, the means of seconds at which participants stopped the videos were converted into % of frames containing the “self”. There is evidence from previous research using brain stimulation that the ability for self-other discrimination is influenced independently of the direction of morphing videos^46–48^. Given that we were not interested in the directions themselves, we averaged across the two directions of morphing (“self to other” and “other to self”) and the mean values were submitted to a 2 × 2 × 2 ANOVA, with the factors of Synchrony (synchronous vs. asynchronous), Stroking velocity (slow vs. fast) and Judgment (pre vs. post). All post hoc analyses were performed using Bonferroni correction. Level of significance was set to 0.05.

### Experiment 2

#### Participants

Thirty-eight right-handed healthy females (age 22.30 ± 3.31SD years) were recruited from UCL Psychology Subject Pool and took part in a single 1-hour session for course credit or £10. Due to a technical problem we were unable to play the morphing videos for one of the participants so we only have subjective ratings for this subject. Participants had no known physical and mental illness and no skin diseases. Signed informed consent was obtained from all participants prior to their participation. The study was approved by the Ethics Committee of the Research Department of Clinical, Educational and Health Psychology, University College London. The experiment was performed in accordance with relevant guidelines and regulations.

#### Design, Materials, Procedure

Design, materials and procedures were identical to Experiment 1, but in this experiment in half of the blocks participants were touched on a congruent location (i.e. cheek) with slow vs. fast velocity, and in the other half they were touched on an incongruent location (i.e. forehead) with slow vs. fast velocity.

#### Data analysis

Data analysis was identical to Experiment 1 but with Congruence used instead of Synchrony as a within-subjects factor.

### Data Availability

The datasets generated during and/or analysed during the current study are available from the corresponding author on reasonable request.

## Electronic supplementary material

Dataset Experiments 1-2

## References

[1] Craig A (2002). How do you feel? Interoception: the sense of the physiological condition of the body. Nature Reviews Neuroscience.

[2] Ceunen, E., Vlaeyen, J. & Van Diest, I. On the Origin of Interoception. *Frontiers in Psychology* **7** (2016). 10.3389/fpsyg.2016.00743 PMC4876111 27242642

[3] Olausson H (2002). Unmyelinated tactile afferents signal touch and project to insular cortex. Nature Neuroscience.

[4] McGlone F, Vallbo A, Olausson H, Loken L, Wessberg J (2007). Discriminative touch and emotional touch. Canadian Journal of Experimental Psychology/Revue canadienne de psychologie expérimentale.

[5] Vallbo ÅB, Olausson H, Wessberg J (1999). Unmyelinated afferents constitute a second system coding tactile stimuli of the human hairy skin. Journal of Neurophysiology.

[6] Liu Q (2007). Molecular genetic visualization of a rare subset of unmyelinated sensory neurons that may detect gentle touch. Nature Neuroscience.

[7] Löken L, Wessberg J, Morrison I, McGlone F, Olausson H (2009). Coding of pleasant touch by unmyelinated afferents in humans. Nature Neuroscience.

[8] Morrison I, Bjornsdotter M, Olausson H (2011). Vicarious Responses to Social Touch in Posterior Insular Cortex Are Tuned to Pleasant Caressing Speeds. Journal of Neuroscience.

[9] Gazzola V (2012). Primary somatosensory cortex discriminates affective significance in social touch. Proceedings of the National Academy of Sciences.

[10] Crucianelli, L., Metcalf, N., Fotopoulou, A. & Jenkinson, P. Bodily pleasure matters: velocity of touch modulates body ownership during the rubber hand illusion. *Frontiers in Psychology* **4** (2013). 10.3389/fpsyg.2013.00703 PMC3792699 24115938

[11] van Stralen H (2014). Affective touch modulates the rubber hand illusion. Cognition.

[12] Lloyd, D., Gillis, V., Lewis, E., Farrell, M. & Morrison, I. Pleasant touch moderates the subjective but not objective aspects of body perception. *Frontiers in Behavioral Neuroscience* **7** (2013). 10.3389/fnbeh.2013.00207 PMC3870280 24391563

[13] Crucianelli, L., Krahé, C., Jenkinson, P. & Fotopoulou, A. Interoceptive ingredients of body ownership: Affective touch and cardiac awareness in the rubber hand illusion. *Cortex* (2017) 10.1016/j.cortex.2017.04.018 28532579

[14] Gallagher S (2000). Philosophical conceptions of the self: implications for cognitive science. Trends in Cognitive Sciences.

[15] Tsakiris M, Haggard P (2005). The Rubber Hand Illusion Revisited: Visuotactile Integration and Self-Attribution. Journal of Experimental Psychology: Human Perception and Performance.

[16] Botvinick M, Cohen J (1998). Rubber hands ‘feel’ touch that eyes see. Nature.

[17] Ehrsson H (2007). The Experimental Induction of Out-of-Body Experiences. Science.

[18] Lenggenhager B, Tadi T, Metzinger T, Blanke O (2007). Video Ergo Sum: Manipulating Bodily Self-Consciousness. Science.

[19] Tsakiris M (2008). Looking for Myself: Current Multisensory Input Alters Self-Face Recognition. PLoS ONE.

[20] Sforza A, Bufalari I, Haggard P, Aglioti S (2010). My face in yours: Visuo-tactile facial stimulation influences sense of identity. Social Neuroscience.

[21] Paladino M, Mazzurega M, Pavani F, Schubert T (2010). Synchronous Multisensory Stimulation Blurs Self-Other Boundaries. Psychological Science.

[22] Povinelli D, Simon B (1998). Young children's understanding of briefly versus extremely delayed images of the self: Emergence of the autobiographical stance. Developmental Psychology.

[23] Gallup G (1970). Chimpanzees: Self-Recognition. Science.

[24] Rochat, P. *Others in mind: Social origins of self-consciousness*. (Cambridge University Press, 2009).

[25] Tong F, Nakayama K (1999). Robust representations for faces: Evidence from visual search. Journal of Experimental Psychology: Human Perception and Performance.

[26] Sugiura M (2014). Neural Mechanism for Mirrored Self-face Recognition. Cerebral Cortex.

[27] Apps M, Tsakiris M (2014). The free-energy self: A predictive coding account of self-recognition. Neuroscience & Biobehavioral Reviews.

[28] Tajadura-Jiménez A, Longo M, Coleman R, Tsakiris M (2012). The person in the mirror: Using the enfacement illusion to investigate the experiential structure of self-identification. Consciousness and Cognition.

[29] Aspell J (2013). Turning Body and Self Inside Out. Psychological Science.

[30] Suzuki K, Garfinkel S, Critchley H, Seth A (2013). Multisensory integration across exteroceptive and interoceptive domains modulates self-experience in the rubber-hand illusion. Neuropsychologia.

[31] de Jong J, Keizer A, Engel M, Dijkerman H (2017). Does affective touch influence the virtual reality full body illusion?. Experimental Brain Research.

[32] Gentsch, A., Crucianelli, L., Jenksinson, P. & Fotopoulou, A. In *Affective touch and the neurophysiology of CT afferents* (Olausson, H., Wessberg, J., Morrison, I. & McGlone, F.ed.) 355–384 (Springer, 2016).

[33] Ciaunica, A. & Fotopoulou, A. In *Embodiment, enaction, and Culture investigating the constitution of the shared world* (Durt, C., Fuchs, T. & Tewes, C.ed.) 173–192 (MIT Press, 2017).

[34] Fotopoulou A, Tsakiris M (2017). Mentalizing homeostasis: the social origins of interoceptive inference – replies to Commentaries. Neuropsychoanalysis.

[35] Meltzoff A (2007). ‘Like me’: a foundation for social cognition. Developmental Science.

[36] Meltzoff A (2007). The ‘like me’ framework for recognizing and becoming an intentional agent. Acta Psychologica.

[37] Rohde M, Di Luca M, Ernst M (2011). The Rubber Hand Illusion: Feeling of Ownership and Proprioceptive Drift Do Not Go Hand in Hand. PLoS ONE.

[38] Abdulkarim Z, Ehrsson H (2015). No causal link between changes in hand position sense and feeling of limb ownership in the rubber hand illusion. Attention, Perception, & Psychophysics.

[39] Longo M, Schüür F, Kammers M, Tsakiris M, Haggard P (2008). What is embodiment? A psychometric approach. Cognition.

[40] Tajadura-Jiménez A, Tsakiris M (2014). Balancing the “inner” and the “outer” self: Interoceptive sensitivity modulates self–other boundaries. Journal of Experimental Psychology: General.

[41] Zeller D, Litvak V, Friston K, Classen J (2015). Sensory Processing and the Rubber Hand Illusion—An Evoked Potentials Study. Journal of Cognitive Neuroscience.

[42] Gentsch A, Panagiotopoulou E, Fotopoulou A (2015). Active Interpersonal Touch Gives Rise to the Social Softness Illusion. Current Biology.

[43] Morrison I (2016). Keep Calm and Cuddle on: Social Touch as a Stress Buffer. Adaptive Human Behavior and Physiology.

[44] Krahé, C., Springer, A., Weinman, J. & Fotopoulou, A. The Social Modulation of Pain: Others as Predictive Signals of Salience – a Systematic Review. *Frontiers in Human Neuroscience* **7** (2013). 10.3389/fnhum.2013.00386 PMC3719078 23888136

[45] Krahé C, Drabek M, Paloyelis Y, Fotopoulou A (2016). Affective touch and attachment style modulate pain: a laser-evoked potentials study. Philosophical Transactions of the Royal Society B: Biological Sciences.

[46] Heinisch C, Dinse H, Tegenthoff M, Juckel G, Brüne M (2010). An rTMS study into self-face recognition using video-morphing technique. Social Cognitive and Affective Neuroscience.

[47] Heinisch C, Krüger M, Brüne M (2012). Repetitive transcranial magnetic stimulation over the temporoparietal junction influences distinction of self from famous but not unfamiliar others. Behavioral Neuroscience.

[48] Payne S, Tsakiris M (2016). Anodal transcranial direct current stimulation of right temporoparietal area inhibits self-recognition. Cognitive, Affective, & Behavioral Neuroscience.

